# Cardiovascular Disease Risk Assessment in the United States and Low- and Middle-Income Countries Using Predicted Heart/Vascular Age

**DOI:** 10.1038/s41598-017-16901-5

**Published:** 2017-11-30

**Authors:** Duke Appiah, Beatrix D. Capistrant

**Affiliations:** 1 Department of Public Health, Texas Tech University Health Sciences Center, Abilene, TX USA; 2 Statistical & Data Sciences, Smith College, Northampton, MA USA; 3 Division of Epidemiology and Community Health, University of Minnesota Minneapolis, Minneapolis, MN USA

**Keywords:** Cardiovascular diseases, Epidemiology

## Abstract

Almost 80% of the global burden of cardiovascular disease (CVD) occurs in low- and middle-income countries (LMICs). However, LMICs do not have well-established, low-technology ways to quantify and communicate CVD risk at population or individual levels. We examined predicted heart/vascular age (PHA) in six LMICs and the United States. Data were from CVD-free adults in World Health Organization Study on Global Aging and Adult Health (n = 29094) and US National Health and Nutritional Examination Survey (n = 6726). PHA was calculated using the non-laboratory Framingham CVD risk equation. High excess PHA (HEPHA) was defined as the differences between PHA and chronological age >5 years. Logistic regression models were used to identify factors associated with HEPHA. Age-standardized prevalence of HEPHA was higher in Russia 52%; China 56%; Mexico 59%; and South Africa 65% compared to the US 45%, Ghana 36%; and India 38%. In LMICs, higher income, being divorced/widowed, alcohol intake and abdominal obesity had higher odds of HEPHA; higher education, fruit intake and physical activity had lower odds of HEPHA. The use of PHA may offer a useful avenue to communicate CVD risk. Interventions tailored at socioeconomic and cultural factors that influence CVD risk factors may be necessary to prevent CVD in LMICs.

## Introduction

Cardiovascular disease (CVD) remains the leading cause of mortality worldwide, resulting in 18 million deaths each year^1^. In 2001, almost 80 percent of all deaths attributable to coronary heart disease occurred in low- and middle-income countries (LMICs)^2^. While the age-adjusted CVD mortality has declined in high income countries over the past decades, the incidence and mortality attributable to CVD has been projected to reach epidemic levels in LMICs by the year 2020, resulting in it being the leading cause of death in these countries^3,4^. A greater cause of concern is that CVD deaths occurs at younger ages in LMICs compared to high income countries^5^.

Nearly 80% of the incidence of cardiovascular diseases worldwide could be prevented with healthier lifestyle and CVD risk factor reduction (smoking, hypertension, diabetes, dyslipidemia and obesity)^6^. Several LMICs do not have well-established, low-technology avenues to quantify and communicate CVD risk at the individual or population levels^7^. Therefore, with very limited resources available for healthcare delivery to effectively treat and manage CVD in LMICs^7^, identifying low-cost strategies to effectively communicate CVD risk with the aim of influencing behavioral change to prevent CVD remain a high priority. The inability to understand the concept of risk has been identified as a reason for the low rate of adoption of heart-healthy behavioral habits among individuals^8,9^, especially those in LMICs^10–12^, Intuitive means of quantifying and communicating CVD risk is particularly important in settings with low health literacy like LMICs.

D’Agostino *et al*.^13^, introduced the epidemiologic concept of predicted heart/vascular age (PHA) which is the predicted age of a person’s cardiovascular system based on their CVD risk factor profile, and presented algorithms for estimating PHA from the landmark Framingham Heart Study. The comparison of PHA to chronological age has been shown to provide a simple, meaningful and easily understood measure of CVD risk compared to the commonly used 10-year absolute risk^14–16^. Accordingly, some emerging evidence suggests that communicating CVD risk by means of PHA is superior in eliciting more emotional impact to adapt healthy lifestyles especially among younger adults at higher levels of CVD risk than the 10-year absolute risk^9^.

Although the PHA might be an intuitive and impactful metric of CVD risk in lower-resourced settings, no studies to date have estimated PHA in LMICs.

The objective of this study was to examine differences in CVD risk factor burden using PHA in LMICs. To put our findings in perspective, we compared estimates of PHA from LMICs to the United States (US), a high-income country. Several factors including socioeconomic changes, urbanization and technological development, social and cultural norms have all been suggested to play a role in CVD risk burden^2,3,17,18^. Therefore, we also assessed the influence of sociodemographic and lifestyle related factors on excess PHA (difference between PHA and chronological age) in LMICs.

## Results

**Figure 1:**
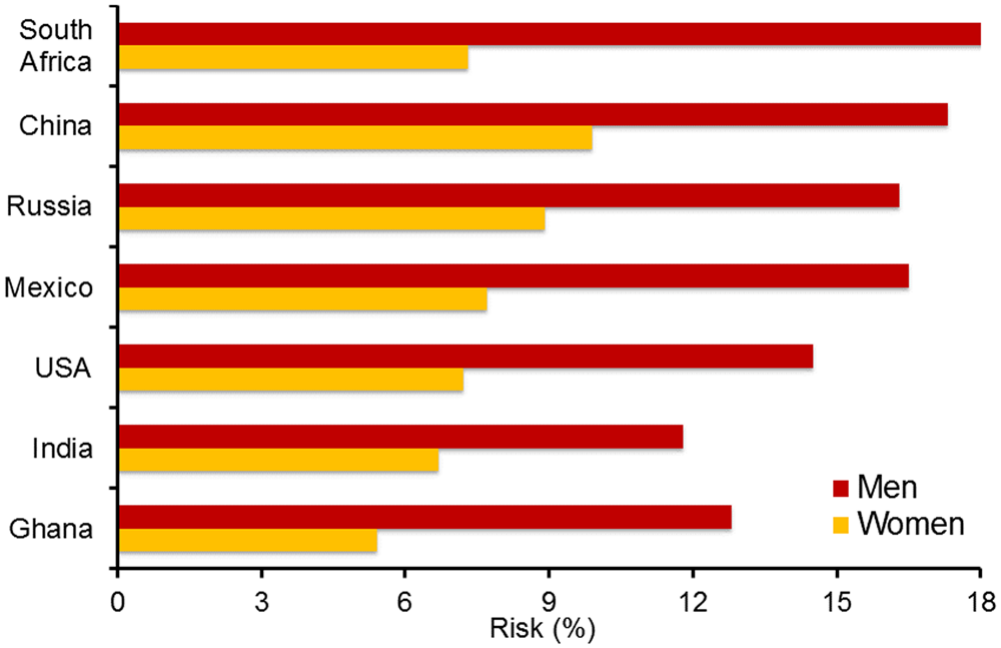
Age-standardized mean 10-year CVD risk among men and women aged 30–74 years by country, SAGE (2007–2010) and NHANES (2007–2010).

Participant characteristics for SAGE and NHANES are reported in Table 1. On average, there were large national variations in adiposity measures with about a third of participants from South Africa (37%), USA (36%) and Mexico (30%) observed to be obese compared to 5.2% and 2.6% of participants from China and India, respectively. The prevalence of self-reported diabetes was lowest in Ghana (1.9%) and highest in Mexico (11.4%). The prevalence of hypertension also varied across countries with South Africa reporting the highest prevalence of 55.3%. A greater proportion of participants from US (25.1%) and Russia (18.1%) reported use of antihypertensive medications compared to only 5.2 of participants from Ghana where the prevalence of hypertension was 45%. The 10-year CVD risk varied substantially across countries (India 9.3%; Ghana 9.4%; US 10.7%; Russia 11.2%; Mexico 12.4%; China 12.8%; and South Africa 13.5%.) and by sex (Fig. 1).

**Table 1:** The distribution of components of the Framingham risk equation among adults aged 30–74 years enrolled in SAGE (2007–2010) and NHANES (2007–2010).

Characteristic	SAGE						NHANES
	China	Ghana	India	Mexico	Russia	South Africa	USA
N	10834	3621	7722	1693	2160	3064	6726
Age, years	46.1 (0.1)	44.8 (0.3)	45.2 (0.2)	43.3 (0.8)	47.1 (1.1)	45.4 (0.7)	48.8 (0.3)
Sex, Female, %	48.8	48.6	45.4	53.4	60.8	49.8	51.1
Current smoker, %	34.1	8.2	48.7	26.8	24.8	27.0	21.0
Body Mass Index, kg/m^2^	23.9 (0.1)	24.4 (0.3)	20.8 (0.2)	28.9 (0.4)	26.8 (0.6)	29.9 (0.6)	28.9 (0.1)
Diabetes, %	2.7	1.9	3.4	11.4	2.6	2.8	11.0
Systolic blood pressure, mmHg	131 (0.9)	130 (0.8)	118 (0.4)	131 (1.4)	129 (2.2)	136 (1.6)	122 (0.3)
Treatment for Hypertension, %	7.9	5.4	6.6	6.7	18.1	8.2	25.1
Obesity, %	5.2	13.1	2.6	30.8	20.9	37.0	35.9
Hypertension, %	40.1	44.7	25.9	32.8	35.3	55.3	32.6

Values are survey weighted mean (standard error) for continuous variables and percentages for categorical variables.

**Figure 2:**
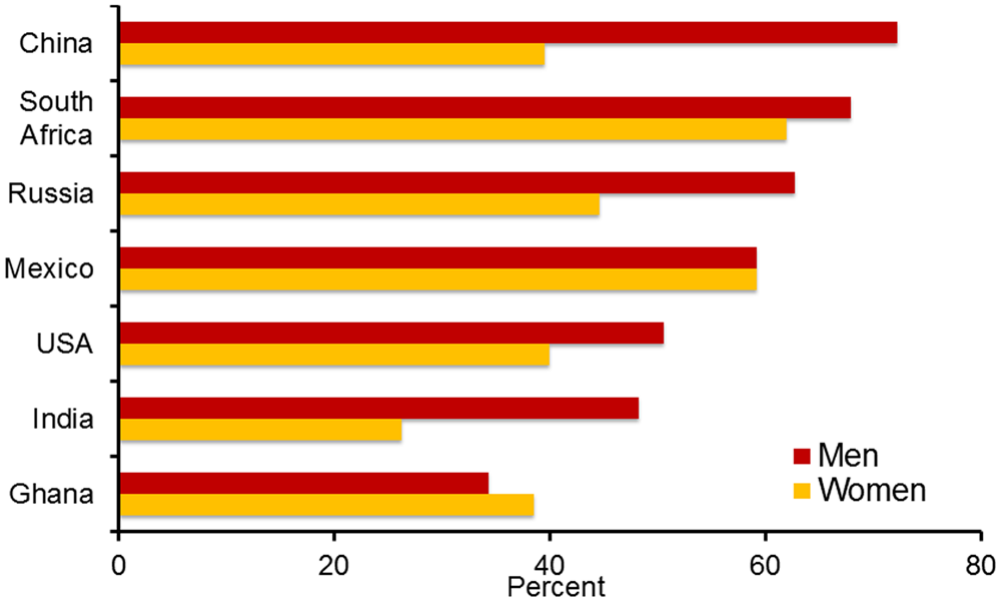
Age-standardized prevalence of high excess predicted heart/vascular age (HEPHA) among men and women aged 30–74 years by country, SAGE (2007–2010) and NHANES (2007–2010).

Similar to the observations for the 10-year CVD risk, there was substantial heterogeneity in the distribution of mean age-standardized PHA, excess PHA and the prevalence of high excess predicted heart/vascular age (HEPHA) between countries. The mean excess PHA was 3.6, 4.2, 7.6, 8.1, 9.5, and 10.5 years for India, Ghana, Russia, China, Mexico, and South Africa, respectively, compared to 6.2 years for the US. The prevalence of HEPHA across countries was as follows: Ghana 36%; India 38%; US 45%; Russia 52%; China 56%; Mexico 59%; and South Africa 65%. Overall, the mean excess PHA was 0.46 years (95% confidence interval (CI): 0.01, 0.90; p = 0.044) higher in LMICs (across all six countries) than the US. Similarly, the prevalence of HEPHA was almost 3% higher in LMICs (across all six countries) compared to the US (2.6%; CI: 0.1, 5.2; p = 0.043). The highest gender disparity in the prevalence of HEPHA was found in China and India (Fig. 2).

**Figure 3:**
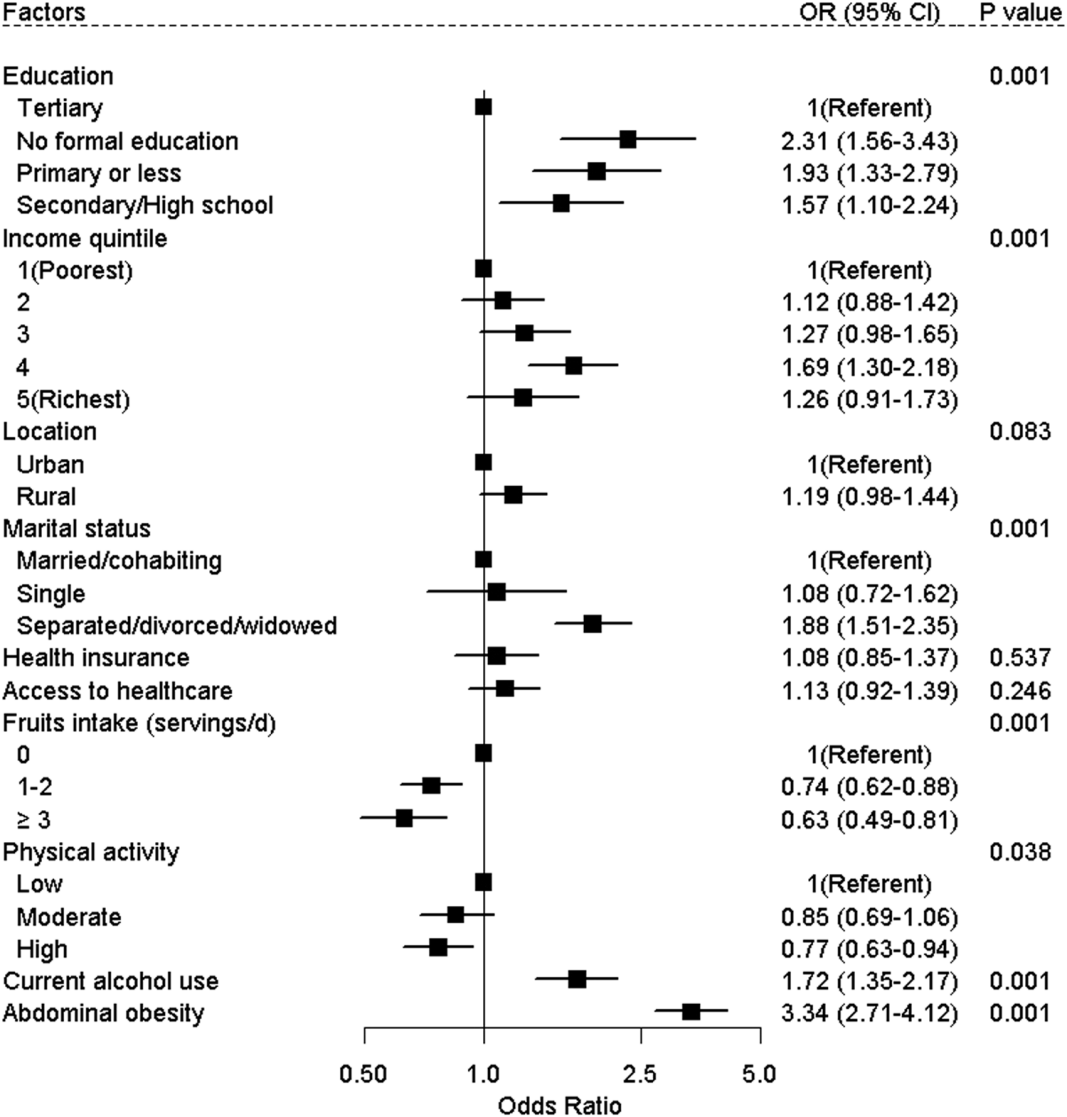
Pooled estimates for the association of sociodemographic and lifestyle factors with high excess predicted heart/vascular age among adults aged 30–74 years, SAGE (2007–2010).

The distribution of socioeconomic, behavior and lifestyle characteristics among the 6 LMIC’s are presented in Table 2. Overall, results from the pooled multivariable adjusted logistic model showed that being separated/divorced/widowed, current alcohol use, having no formal education or fewer years of education, and having higher income were all associated with higher odds of HEPHA (Fig. 3). Notably, central or abdominal obesity was associated with a threefold higher odds of HEPHA (OR = 3.34, CI: 2.71, 4.12). A higher daily intake of fruits and high physical activity levels were inversely associated with HEPHA. These associations were fairly consistent across all countries, however, interaction analyses revealed few exceptions (Table 3). We failed to find an association of vegetable intake, location, and access to healthcare with HEPHA in either pooled or country-stratified models.

**Table 2:** The distribution (survey weighted percentages) of socioeconomic and lifestyle/behavior factors among adults aged 30–74 years by country, SAGE (2007–2010).

Characteristic	China	Ghana	India	Mexico	Russia	South Africa
N	10834	3621	7722	1693	2160	3064
Education, %						
No formal education	7.2	26.5	39.1	5.7	0.1	9.8
Primary or less	31.6	31.9	26.2	51.4	1.0	31.4
Secondary/High school	53.8	37.1	27.3	31.3	76.7	51.5
Tertiary	7.3	4.5	7.4	11.5	22.2	7.3
Income/wealth quintile, %						
1 (Poorest)	9.7	13.6	21.9	13.4	9.6	15.8
2	16.6	17.8	21.5	24.3	9.9	24.0
3	19.2	19.6	19.6	21.2	13.3	20.3
4	24.3	22.2	18.4	15.6	26.8	21.5
5 (Richest)	30.3	26.7	18.6	25.4	40.4	18.3
Location, %						
Urban	46.0	46.4	24.6	75.3	81.2	69.9
Rural	54.0	53.6	75.4	24.7	18.8	30.1
Marital Status, %						
Single	1.4	4.1	1.6	13.1	3.9	18.9
Married/Cohabiting	94.3	76.9	88.9	79.5	70.7	62.4
Separated/divorced/widowed	4.3	19.0	9.5	7.5	25.3	18.7
Health Insurance, %	85.5	29.4	3.5	59.1	99.5	25.8
Health care access, %	71.7	79.2	84.5	87.0	80.9	76.4
Fruits intake (servings/d), %						
0	20.8	9.5	42.0	10.0	9.5	15.0
1–2	32.0	61.0	53.8	63.3	70.1	58.8
≥3	47.1	29.5	4.2	26.7	20.4	26.2
Current alcohol use, %	27.5	32.2	11.3	31.9	57.0	18.3
Abdominal obesity, %	14.1	24.6	11.4	49.3	26.5	37.3
Physical activity, %						
Low	29.9	21.3	15.2	31.6	13.9	53.1
Moderate	28.3	18.8	16.9	32.3	24.7	18.1
High	41.7	59.9	67.9	36.1	61.4	28.8

**Table 3:** Country-specific odds ratios for the association of socioeconomic and lifestyle factors with HEPHA among adults aged 30–74 years, SAGE (2007–2010).

Characteristic	China	Ghana	India	Mexico	Russia	South Africa
N	10834	3621	7722	1693	2160	3064
Education						
Tertiary	1 Referent	1 Referent	1 Referent	1 Referent	1 Referent	1 Referent
No formal education	3.88 (2.09–7.18)	1.49 (0.59–3.76)	2.12 (1.32–3.39)	3.47 (0.88–13.7)	0.53 (0.05–5.27)	5.45 (1.34–22.1)
Primary or less	2.36 (1.53–3.63)	1.08 (0.44–2.68)	1.84 (1.16–2.91)	1.22 (0.39–3.83)	13.1 (4.00–42.9)	2.82 (0.76–10.5)
Secondary/High school	1.95 (1.38–2.76)	0.84 (0.34–2.09)	1.43 (0.91–2.26)	1.03 (0.32–3.26)	1.26 (0.50–3.15)	1.86 (0.53–6.52)
Income/wealth quintile						
1 (Poorest)	1 Referent	1 Referent	1 Referent	1 Referent	1 Referent	1 Referent
2	1.02 (0.65–1.60)	0.60 (0.35–1.02)	1.38 (1.00–1.92)	4.61 (1.39–15.3)	0.57 (0.24–1.38)	0.94 (0.35–2.49)
3	1.04 (0.64–1.69)	1.33 (0.82–2.16)	1.47 (1.06–2.05)	1.39 (0.44–4.36)	0.62 (0.27–1.47)	3.27 (1.21–8.83)
4	1.50 (0.94–2.39)	0.99 (0.59–1.68)	1.77 (1.27–2.47)	1.84 (0.66–5.18)	1.40 (0.58–3.37)	2.31 (0.77–6.94)
5 (Richest)	1.28 (0.72–2.25)	0.88 (0.50–1.56)	2.04 (1.42–2.93)	2.39 (0.76–7.57)	0.22 (0.09–0.51)	4.99 (1.66–14.9)
Location (Rural)	1.32 (0.95–1.85)	0.75 (0.53–1.07)	0.89 (0.71–1.12)	0.70 (0.30–1.65)	1.09 (0.58–2.06)	1.27 (0.56–2.88)
Marital Status						
Married/Cohabiting	1 Referent	1 Referent	1 Referent	1 Referent	1 Referent	1 Referent
Single	1.32 (0.71–2.45)	1.46 (0.58–3.67)	0.67 (0.35–1.29)	0.51 (0.23–1.13)	0.28 (0.10–0.78)	1.57 (0.66–3.72)
Separated/divorced/widowed	2.86 (2.10–3.89)	1.75 (1.18–2.59)	1.68 (1.25–2.26)	2.33 (0.87–6.24)	1.06 (0.62–1.81)	0.76 (0.33–1.75)
Health Insurance	1.26 (0.93–1.70)	1.00 (0.70–1.44)	1.37 (0.93–2.02)	0.80 (0.46–1.40)	1.45 (0.24–8.59)	0.13 (0.06–0.29)
Health care access	1.19 (0.87–1.62)	0.84 (0.57–1.23)	0.92 (0.72–1.17)	1.16 (0.41–3.32)	1.52 (0.62–3.70)	1.37 (0.64–2.91)
Fruits intake (servings/d)						
0	1 Referent	1 Referent	1 Referent	1 Referent	1 Referent	1 Referent
1–2	0.77 (0.52–1.13)	1.07 (0.64–1.78)	0.75 (0.62–0.90)	1.34 (0.37–4.83)	0.32 (0.15–0.68)	0.97 (0.43–2.21)
≥3	0.58 (0.40–0.84)	1.02 (0.58–1.79)	0.93 (0.60–1.46)	1.37 (0.34–5.49)	0.47 (0.17–1.29)	0.56 (0.21–1.51)
Current alcohol use	1.49 (1.07–2.09)	1.26 (0.92–1.72)	2.36 (1.71–3.26)	0.53 (0.25–1.12)	1.28 (0.63–2.58)	2.90 (1.22–6.89)
Abdominal obesity	3.76 (2.85–4.96)	2.95 (1.93–4.52)	2.60 (2.08–3.26)	2.23 (1.18–4.23)	3.00 (1.51–5.97)	3.98 (1.99–7.98)
Physical activity						
Low	1 Referent	1 Referent	1 Referent	1 Referent	1 Referent	1 Referent
Moderate	0.83 (0.63–1.10)	0.64 (0.41–1.00)	1.00 (0.76–1.34)	1.22 (0.53–2.80)	0.73 (0.31–1.74)	0.55 (0.23–1.28)
High	0.81 (0.59–1.11)	0.70 (0.47–1.05)	0.85 (0.67–1.08)	1.18 (0.57–2.43)	0.60 (0.30–1.21)	0.94 (0.41–2.15)

## Discussion

In this study of seven countries from 4 continents, we observed that CVD risk burden as assessed by PHA among adults aged 30–74 years was substantially higher than their chronological age. However, there were substantial variations in this excess PHA among countries; overall, it tended to be slightly higher in LMICs than the US. Furthermore, among LMICs, higher income, none or fewer years of education, being separated/divorced/widowed, alcohol use, and abdominal obesity were all associated with higher odds of HEPHA, while daily intake of fruits and high physical activity were associated with lower odds of HEPHA. To our knowledge, this is the first study to provide population-level estimates of the burden of CVD risk factors using objectively measured risk factors to estimate excess PHA across multiple countries with emphasis on LMICs where a greater proportion of the global CVD mortality occur.

Prior studies using PHA or “cardiovascular age”, “vascular age”, or “cardiovascular age risk” to communicate risk have largely been conducted in specific ethnic groups^19^, disabled populations^20^ and in clinical settings among persons with low-to-moderate^21^ or high risk^22–25^ for CVD, as well as patients with morbid conditions such as schizophrenia^26,27^, human immunodeficiency virus^28–30^, hepatitis C virus^28^ and cancer^31^. In all these studies, the Framingham risk equation was the most common model used to calculate PHA, followed by the Systematic Coronary Risk Evaluation (SCORE) equation. While PHA was found to be higher than chronological age in all these studies, the difference between PHA and chronological age (excess PHA) varied from 2 to 26 years^16^. The few studies reporting country-level estimates for PHA or excess PHA have mainly come from developed or high income countries. A web-based health assessment study using heart age to communicate CVD risk among 2.7 million first-time users aged 21–80 years who had no prior coronary disease in 2009–2011 from 13 countries (United Kingdom, Germany, the Netherlands, Belgium, Finland, Austria, Poland, Turkey, Ireland, Portugal, Slovenia, Greece and Australia) reported a mean excess PHA of 4.9 and 3.3 among men and women, respectively^32^. The high proportion of participants in the web-based study who were unaware of their systolic blood pressure (47%) and other physiological risk factor values led to overestimation of PHA by an average 2.1–4.5 years^32^. Furthermore, no attempts were made to compare excess PHA across among the 13 countries. Using data from the 2011 and 2013 US Behavioral Risk Factor Surveillance System (BRFSS), Yang *et al*.^14^, reported an average excess PHA of 7.8 and 5.4 years among adult men and women aged 30–74, respectively. These results were higher than the estimates obtained from the present study for the US (men: 7.7, women: 4.8). This discrepancy could be due to the BRFSS study using a model-estimated systolic blood pressure instead of measured systolic blood pressure and not also excluding persons with prevalent cardiovascular disease which could all have contributed to the higher excess PHA observed.

A major factor that influences motivation to adapt healthy lifestyles to improve cardiovascular health is the individual’s perception of CVD risk^9^. For instance, among current smokers, those who perceived themselves to be at high risk for stroke were observed to be more likely to participate in stroke prevention initiatives compared to those who perceived their risk of stroke to be low^8^. Most CVD risk prediction algorithms which estimate absolute risk are not easily understood by non-scientists which results in many individuals underestimating their risk especially women^33,34^. Regardless of age, CVD risk expressed as PHA has been shown to be more accurately recalled than absolute risk (expressed as percent)^35^. PHA is superior in communicating risk than 10-year absolute risk, and it elicits more emotional impact to adapt healthy lifestyles especially among younger adults at higher levels of CVD risk^9,36^. This observation among young adults is of great importance since CVD risk is greatly influenced by age. More often than not, young adults with poor cardiovascular health tend to have low 10-year absolute risk scores and this may not motivate them to embrace lifestyles and behavior changes that will improve their cardiovascular health^16^. Lopez-Gonzalez *et al*.^9^, in their single-blind randomized intervention trial conducted in Spain among 3153 public sector workers observed a greater change in anthropometric and metabolic parameters after 1 year in persons who had their CVD risk communicated to them as PHA than 10-year absolute risk at baseline. This improved change in anthropometric and metabolic parameters in persons who had their CVD risk communicated to them as PHA at baseline was translated into greater improvements in 10-year absolute risk score (−0.4%) and PHA values that were on average 1.5 years younger than at baseline. Although the group that had their CVD risk communicated to them as 10-year absolute risk also saw some improvement in cardiovascular health, thus a 0.2% reduction in 10-year absolute risk score and 0.3 years difference in PHA at baseline and 1 year, it was markedly less than the improvements recorded for the PHA group. This, together with other observations have prompted some medical organizations in high income countries such as the Canadian Cardiovascular Society^37^ and the Joint British Societies to recommend, in their clinical guidelines, the use of PHA to communicate lifetime risk and in some cases, inform pharmacological therapy decision making^38^.

In developing countries, the transformations in health care infrastructure or advanced treatment procedures are struggling to catch up with the accelerated rate of adverse CVD risk factor associated with the socioeconomic transformation that these nations are going through. With the management of behavioral/lifestyle related CVD risk factors at the individual and population level lying outside of the domain of the healthcare systems in many LMICs^2^, it is refreshing for public health efforts that some studies have observed PHA to offer a useful avenue to communicate CVD risk and motivates heart-healthy lifestyle changes even without clinician involvement^16,22,39^.

Since some of the CVD risk factors (glucose and blood pressure) required for estimating PHA are often assessed in the clinical setting^40^, the use of PHA at the population level may be limited. However, emerging evidence has shown that in LMICs, it is feasible to incorporate blood pressure monitoring and glucose measurements in community-based screening programs for infectious disease^41,42^. Using inexpensive instruments such as the paper-based glucose tests in these targeted community screenings and interventions can further enhance easy ascertainment of diabetes^43^, an important component of PHA. This will further aid in accurate measurement of CVD risk factor burden. Alternatively, PHA ascertainment could be included in some of the lay volunteer-led, culturally adapted community-based CVD risk factor screening programs which are already in existence^44^. To effectively implement interventions tailored at improving some of the behavior and socioeconomic (or cultural factors) identified to be associated with PHA in the present study will require policy and system changes in LMICs.

Results from the present study showed income to be positively associated with higher odds of CVD risk factor burden in some LMIC’s. However, the reverse was seen in Russia where higher income was associated with lower odds of HEPHA, consistent with findings from the Mexican Health and Aging Study^45^ and The International Clinical Epidemiology Network^46^. These diverging results on the influence of income on CVD risk expand the evidence that the socioeconomic setting of a country influences the association of socioeconomic status with CVD risk factor burden or disease at the individual level^47–49^. For instance, at the time of SAGE wave 1, Russia was well advanced in its economic transition than the other countries and thereby observed a socioeconomic-CVD relationship consistent with those reported for developed countries unlike the other 5 LMICs. In most LMICs, it is the wealthy that are first to adopt a Western lifestyle due to wealth and exposure^50^; indeed, CVD risk is often conceptualized as a reverse social gradient compared to what has been seen in the US^51,52^. We speculate that the observed positive association between income and HEPHA may be explained, in part, by the definition of income employed by SAGE which was not only based on salary but on household dwelling characteristics, ownership of selected assets and access to basic services.

The strengths of this study include the use of large nationally representative samples of adults from various regions of the world. Additionally, a standardized study design and sampling methods was used across all 6 countries which enabled cross-country comparisons to be undertaken. Potential limitations of this study warrant consideration. First, response rates varied across countries, and in countries where response rate was low, the potential for selection bias cannot be excluded. Second, there were some methodological differences in measures of CVD risk factors between SAGE and NHANES. For example, physician diagnosis of diabetes status were self-reported and may have been underestimated in some countries in SAGE due to differential accessibility to healthcare, compared to the US. In resource-limited settings where individuals usually tend to seek healthcare when they are symptomatic or have advanced stage of disease^53^, the possibility of under-reporting of diabetes cannot be ruled out. This may explain the low prevalence of diabetes observed in Ghana. However, the reported estimate is similar to the current country prevalence reported by International Diabetes Federation^54^. Furthermore, in SAGE, blood pressure was obtained using a wrist monitor while brachial monitors were used in NHANES. Wrist monitors have been reported to be more prone to inaccuracy than brachial monitors^55^. However, wrist monitors have been reported to be reliable as long as the measurement device is positioned at heart level^56^. In some developing countries, wrist monitors have been found to provide measurements that are consistent with brachial monitors which in some cultures may be inappropriate to use, especially for women^57^. Additionally, the use of an average of all available blood pressure measurements in SAGE may overestimated systolic blood pressure values, primarily due to white coat reactions^55^. Since blood pressure measurements were taken in the homes of participants, coupled with no material differences in the mean blood pressure readings regardless of whether the average of the second and third reading or all available readings were used, any bias in blood pressure measurements, if present, may be very minimal. Third, apart from the Framingham risk equation, it would be ideal to compare the PHA with other CVD risk equations like the pooled cohort equation in assessing CVD risk burden in LMICs. However, we were unable to perform such analysis since most CVD risk equations, including the pooled cohort equation, require some clinical markers (HDL and total cholesterol) which are not currently available for the SAGE study, and to the best of our knowledge, no non-laboratory algorithm for the pooled cohort equation are currently available. Unless in a clinical setting where biochemical measures can be used to calculate CVD risk, the use of PHA offers a best avenue for CVD prevention among people who do not have access to adequate healthcare or those who refuse to seek medical attention until the advanced stages of disease. Finally, it is possible that the Framingham risk equation used to calculate PHA may have overestimated the risk for CVD despite it being validated in several regions of the world^58^. While the present study illustrate the usefulness of the PHA, any potential bias in this estimate should not influence an individual’s perception of their own risk. In this light, we believe that the current study provides an avenue, even if not entirely perfect, to improve primary prevention of CVD in low-and-middle income countries who bear the greatest global burden of CVD events through effective communication of CVD risk using PHA.

In conclusion, CVD risk burden as assessed by PHA varied substantially across countries with participants having predicted heart/vascular age that were significantly higher than their chronological age, especially among individuals from LMICs. This study contributes new and substantial information to the literature and also holds potential to stimulate public health support for implementing policies and programs targeting socioeconomic and behavior lifestyle modification in LMICs at the individual and population level to improve cardiovascular health. Future studies that aim to estimate the influence of lifestyle changes that support cardiovascular health such as smoking cessation, increased physical activity and weight loss especially in the abdominal region on changes in PHA may contribute valuable evidence to motivate individuals to adapt heart-healthy lifestyles.

## Methods

### Predicted Heart/vascular age

PHA was calculated based on the non-laboratory Framingham risk score equation for general CVD developed by D’Agostino *et al*.^13^ which is recommended for use in resource-limited settings. The risk equation includes age, sex, systolic blood pressure, treatment for hypertension, current smoking and diabetes status, and body mass index (BMI). Although PHA is obtained from the same non-laboratory Framingham equation that is used to obtain the 10-year CVD risk, PHA facilitates easier understanding than the concept of risk. In other words, PHA is a person’s CVD risk transformed into the units of age rather than risk. Specifically, to the age of a person with the same risk but all other risk factors at the normal level. For example, using the example provided by D’Agostino *et al*.^13^, if a 61-year-old woman with risk factors above normal levels has a PHA score of 73, she has the heart age/vascular age equivalent to a 73-year-old woman with normal risk factors. This metric’s intuitive interpretation – that your heart is 12 years older than you are – has useful clinical and public health properties for use in lower resourced settings. Accordingly, excess PHA was calculated as the difference between predicted heart/vascular age and chronological age. A positive value means that PHA exceeds biological age which indicates higher CVD risk and a greater burden of CVD risk factors. Similar to previous studies^14^, we estimated high excess predicted heart/vascular age (HEPHA) as excess PHA exceeding 5 years.

### The World Health Organization Study on Global Aging and Adult Health (SAGE)

The study design and participant recruitment for SAGE have been previously described^59^. Briefly, SAGE is a multi-country longitudinal study which used a multistage stratified random sampling strategy to select non-institutionalized adults aged ≥18 years from six LMICs (China, Ghana, India, Mexico, Russia and South Africa). In each country, adults aged 50 years or older were oversampled. The response rates by country for this population-based household and individual survey were 93% (China), 83% (Russia), 80% (Ghana), 77% (South Africa), 68% (India), and 51% (Mexico). All participants provided written informed consent with data collection protocols approved by the Institutional Review Boards of each country as well as the World Health Organization (WHO) Ethical Review Committee.

### Measures

Trained interviewers, using standardized survey instruments, carried out face to-face interviews with participants to obtain information on demographics (age, sex, marital status), socio-economic factors, behavioral/lifestyle factors and medical conditions which were all self-reported. Education was assessed based on the highest level of education completed and harmonized across countries using international standards^60^. Income index was calculated based on household dwelling characteristics, ownership of selected assets and access to basic services^61^. Country-specific quintiles were then generated with the lowest quintile indicating lowest economic status (poorest). Information on healthcare access was obtained by asking participants whether they were able to get health care the last time they were in need of it. Locations was defined as the primary residence of participants and classified as either urban or rural based on the World Bank standard definitions^62^. Smoking status was assessed by asking participants if they have ever smoked tobacco or used smokeless tobacco. Participants who responded in the affirmative and were currently using tobacco products were considered to be current smokers. Current alcohol use was defined as consuming a drink that contains alcohol (such as beer, wine, spirits, etc.) in the previous 30 days. A 24-hour dietary recall questionnaire was used to ascertain the number of servings of fruits and vegetables typically eaten in a day. In an attempt to standardize the serving size and number of servings reported by participants across countries, a nutrition card with pictures and writings indicating general categories, amounts, and examples of servings of fruits and vegetables was used^63^. The duration, frequency and intensity of physical activity over the past week in three domains: occupational; transport-related; and recreational or leisure time, was assessed using the Global Physical Activity Questionnaire with conventional cut-offs used to categorize participants into low, moderate, or high physical activity levels^64^.

During the survey, participants also underwent a physical examination where height and body weight were measured using a stadiometer and an electronic weighting scale, respectively. BMI was calculated by dividing weight in kilograms by height in meters squared. Obesity was defined as BMI ≥ 30 kg/m^2^. Waist circumference was measured at the navel level using an inelastic tape. Central/abdominal obesity was defined as a waist circumference greater than 102 cm in men and 88 cm in women^65^. Blood pressure was measured three times (1 minute between each measurement) using Medistar Wrist Blood Pressure Model S with participants seated with the arm level to the heart. The mean of all available blood pressure measurements were used for this analysis. For description, hypertension was defined as a mean systolic blood pressure above 140 mmHg, diastolic blood pressure of above 90 mmHg, a physician diagnosis of hypertension, or current medication use for elevated blood pressure. Participants who responded positively to the question of a physician diagnosis of diabetes (high blood sugar) which did not occur during pregnancy (for women) were classified as having diabetes. A diagnosis of angina pectoris was based on self-report and the symptom-based algorithms of the Rose questionnaire^66^.

Overall 47443 adults participated in SAGE wave 1(2007–2010) with 44094 of them partially or fully completing the interviews. The present analysis was restricted to 36165 participants aged 30–74 years (the age limits for the Framingham risk score equation). From this number, participants with prevalent CVD defined as angina or stroke (n = 4275) or those with missing values for covariates used in calculating PHA (n = 2720) were excluded. Since this definition of CVD may miss some participants with the disease (i.e. heart failure, etc. not assessed in SAGE), participants who had inpatient hospital stays of one or more days in the previous 12 months for any heart related condition (n = 76) were excluded to limit misclassification. This resulted in an analytic sample of 29094 participants included in the current analysis.

### National Health and Nutrition Examination Survey (NHANES)

NHANES is a stratified, multistage, probability cluster survey conducted in the non-institutionalized U.S. population that has been well-documented elsewhere^67^. NHANES was approved by the National Center for Health Statistics (NCHS) Institutional Review Board in accordance with the Declaration of Helsinki, and all participants in NHANES had written informed consent prior to the study. To make comparisons of PHA to SAGE wave 1, we selected respondents who participated in the NHANES 2007–2010 surveys. The response rates for the 2007–2008 and 2009–2010 survey years were 78.4% and 79.4%, respectively^68^. Age, sex, current smoking status and high blood pressure medication use were all self-reported. Height and body weight were measured using standardized procedures. BMI and hypertension were defined as previously described^69^ and these were similar to how they were defined in SAGE. Diabetes was defined by one or more of the following: a self-reported physician diagnosis, elevated fasting glucose levels ≥126 mg/dL, oral glucose tolerance test ≥200 mg/dL, or reported use of diabetes medications^70^. We limited the analysis to 6726 adults aged 30–74 years without CVD (self-reported angina, stroke or myocardial infarction) who had complete information for the covariates used in estimating PHA.

### Statistical analysis

We calculated age-standardized mean PHA, excess PHA and prevalence of HEPHA by country using the United Nations population for the year 2010 (https://esa.un.org/unpd/wpp/) as the standard population. Mean and frequencies were calculated to describe characteristics of the sample by country. Among LMICs, logistic regression models were used with odds ratios (OR) estimated to identify socioeconomic, lifestyle and behavior factors associated with the prevalence of HEPHA. To control for unobserved differences among countries such as cultural factors that may influence some behaviors and lifestyle (i.e. nutrition), we included dummy variables for country in all regression models that calculated pooled estimates. Additionally, to obtain estimates independent of the influence of the potential differences in sociodemographic, economic, and lifestyle/behavior factors between men and women, we also adjusted for sex in all models. Factors which were associated with HEPHA (at an alpha level of 0.2) in country and sex adjusted models were included in a final multivariable logistic regression model. In secondary analyses, we performed formal tests for interactions of socioeconomic or lifestyle factors with country and sex. Since no interactions by sex were observed, only pooled and country-specific estimates were reported. All analyses incorporated the complex survey design and sample weights to generate nationally-representative estimates.

### Data availability

This article uses data from WHO Study on Global Ageing and Adult Health (SAGE) Wave 1 which are available at http://www.who.int/healthinfo/sage/cohorts/en/index2.html. Data from the National Health and Nutrition Examination Survey (NHANES) are publicly available through the Centers for Disease Control and Prevention: http://www.cdc.gov/nchs/nhanes/nhanes_questionnaires.htm
